# Responsible research evaluation: integrating quality, leadership, and integrity in national systems. The case of Peru

**DOI:** 10.3389/frma.2026.1842222

**Published:** 2026-06-08

**Authors:** Pablo Alejandro Millones-Gómez, Jean Paul Simon Castillo-Nunez, Kenyie Jossuet Paucca-Calla, Carlos Alberto Minchón-Medina, Vanessa Lizet Castro-Delgado, Samantha Sotelo-Llancari, Cecilia Ignacio-Punin, David Yeret Rodríguez-Salazar, Víctor Hugo Urrutia-Baca

**Affiliations:** 1 Grupo INSCIENCE, Vicerrectorado de Investigación, Universidad Privada Norbert Wiener, Lima, Peru; 2 Faculty of Physical Sciences and Mathematics, Department of Statistics, National University of Trujillo, Trujillo, Peru; 3 Escuela de Ingeniería y Ciencias, Tecnológico de Monterrey, Monterrey, Mexico

**Keywords:** academic leadership, RENACYT, research evaluation, research integrity, scientific production

## Abstract

**Introduction:**

National research evaluation systems often rely on publication-based metrics that equate productivity with performance while overlooking scientific leadership and research integrity. This study examines the Peruvian National Registry of Science, Technology, and Innovation (RENACYT) to inform a more multidimensional framework for research evaluation.

**Materials and methods:**

An observational, non-experimental, and analytical study was conducted using data from RENACYT, Scopus, and SciVal for 9,651 researchers during 2019–2024. Four dimensions were assessed across hierarchical levels: scientific production, journal-based impact (Q1–Q4), corresponding authorship as a proxy of leadership, and retractions as indicators of research integrity. Descriptive statistics, ANOVA, repeated-measures tests, and count regression models (Poisson, negative binomial, and zero-inflated specifications) were applied.

**Results:**

A total of 92,284 publications were identified. Productivity increased across RENACYT levels (3.9 publications in Level VII vs. 62.5 in Distinguished; *F* = 1,162.572, *p* < 0.001), although with substantial within-level dispersion and differentiated temporal trajectories (Time × Level: *F* = 44.662, *p* < 0.001). Higher levels concentrated Q1 output (30.8 vs. 0.7 articles per author; *F* = 1,090.183, *p* < 0.001), while differences became less pronounced in Q3–Q4 journals. Corresponding authorship increased with level (β = 1.624 for Level I, *p* < 0.001) but remained heterogeneous even among top categories. Retractions were positively associated with productivity (coef. = 0.013, *p* < 0.001) reflecting differential exposure to integrity-related risks rather than uniform patterns across levels.

**Conclusion:**

RENACYT captures gradients in productivity and quality but insufficiently differentiates leadership and integrity. These findings support the proposal of a hybrid evaluation framework integrating productivity with explicit recognition of intellectual leadership and research integrity.

## 1 Introduction

Research evaluation has become a defining element in shaping the global scientific ecosystem, influencing academic trajectories, institutional strategies, and national science policies. Beyond its coordinating function, evaluation frameworks also generate unintended consequences by restricting academic autonomy, encouraging opportunistic behavior, and standardizing scholarly activity into administrative conformity (Jin and Jiang, 2024; Lambovska, 2023). To address these tensions, contemporary agendas emphasize integrating both scientific and social value, combining quantitative metrics with qualitative assessments to capture the co-production of knowledge and its societal outcomes (Smit and Hessels, 2021; Thomas et al., 2020). Within this landscape, performance logics such as university rankings have gained prominence, although they remain subject to methodological biases and technical limitations that call for more transparent and context-sensitive practices (Kochetkov, 2024; López-Castellano, 2025). The institutionalization of “impact” as a central category of evaluation has further reshaped academic discourse, reinforcing the need to balance public utility, epistemic quality, and diversity of contributions in knowledge production (Shu et al., 2022; Wróblewska, 2021).

In this context, research evaluation cannot be reduced to isolated indicators, but must be understood as the interaction between multiple dimensions, particularly scientific production (volume of publications), impact (operationalized here through journal quartiles as a proxy of impact and visibility), leadership (authorship roles such as corresponding author), and integrity (approached through retractions as observable outcomes of problematic publication processes). These dimensions are not independent: the pressure to increase production may affect the distribution of publications across journal quartiles, leadership structures influence both productivity and research direction, and integrity—although imperfectly captured through retractions—reflects how incentive systems shape academic behavior and exposure to risk (Ab-Rahman et al., 2011; Fiala et al., 2017; Ramos, 2018; Rauniar and Cao, 2025). Understanding these interdependencies is essential for designing evaluation systems that do not merely measure performance, but actively shape the conditions under which knowledge is produced.

For decades, quantitative indicators such as publication counts and journal impact factors dominated evaluation practices, frequently serving as proxies for quality and influence. However, these reductionist approaches have been increasingly criticized for fostering perverse incentives and distorting academic behavior. The San Francisco Declaration on Research Assessment (DORA) explicitly rejected the use of journal-based metrics for evaluating individual researchers, urging instead that research be judged on its own merits (Cagan, 2013). Similarly, the Leiden Manifesto articulated ten principles advocating for transparent metrics, sensitivity to context, and alignment with institutional missions, providing a roadmap to mitigate the risks of indicator misuse (Hicks et al., 2015). While these statements successfully catalyzed a global critique and established a crucial ethical consensus, their role was largely discursive, dismantling flawed metrics and laying the philosophical foundations for change. Yet this achievement exposed a subsequent challenge: bridging the gap between principles and practice. Attention has thus shifted from conceptual critique to the complex task of operationalizing these principles into tangible and scalable systems.

In recent years, the debate has moved from questioning traditional indicators to implementing structural reforms. The Coalition for Advancing Research Assessment (CoARA) represents a global initiative to rethink evaluation practices by promoting qualitative assessment, narrative curricula vitae, and recognition of diverse contributions to science (CoARA, 2024). National systems such as the Research Excellence Framework (REF) in the United Kingdom, the Valutazione della Qualità della Ricerca (VQR) in Italy, and the Excellence in Research for Australia (ERA) illustrate attempts to balance metrics with peer review, field-normalized indicators, and measures of societal impact. These models, however, are not mere implementations of earlier manifestos but rather more complex second-generation evolutions. They confront the practical difficulties of scaling qualitative judgments, defining “impact” without reintroducing opportunities for manipulation, and managing the high costs of peer review. As such, they are best understood as large-scale experiments in the search for balance between objectivity and expert judgment, highlighting that the transition from theory to practice is iterative and constantly evolving.

At the same time, evaluation systems decisively shape academic behavior and productivity, though their effects vary by context. Researchers face growing pressure to publish in peer-reviewed journals under the “publish or perish” paradigm, with research participation shaped by factors operating at individual, institutional, and national levels (Rhein and Nanni, 2023). National evaluation frameworks directly affect publishing practices, particularly among doctoral candidates and early-career researchers, who adapt their strategies to align with evaluation requirements (Rowlands and Wright, 2021). However, the connection between national policies and actual performance improvements remains weak, as international collaboration appears to be the main driver of national scientific impact rather than domestic reforms (Adams and Szomszor, 2024). Moreover, performance-based funding schemes may generate unintended consequences, as seen in Italy, where selective funding tied to national evaluations proved problematic due to regional disparities in scientific performance and significant variation within institutions (Grisorio and Prota, 2020).

Despite these international advances, challenges persist. Numerous studies warn that equating productivity with quality can lead to fragmented publications, inflated co-authorship, or strategic citation practices that undermine scientific integrity (Fire and Guestrin, 2018; Sandström and van den Besselaar, 2016). Editorial analyses also show that the unchecked growth of publications threatens to overwhelm peer review systems and dilute standards of rigor (Montazerian and Dorch, 2022). The inappropriate use of bibliometric indicators in institutional rankings and funding allocation has further entrenched a culture of quantity over content (Quaderi, 2023). These dynamics illustrate how production, impact (as reflected in quartile distribution), leadership and retractions are structurally interconnected, as pressures to maximize output may simultaneously reshape authorship practices and increase exposure to publication risks, particularly in systems that rely on narrow quantitative criterio (De Albuquerque, 2009; Gundersen, 2017; Ramos, 2018; Rosenthal, 2017; Rost et al., 2024). These risks are not evenly distributed worldwide: researchers in emerging scientific systems are particularly vulnerable to policies that emphasize numerical accumulation without safeguards for quality or integrity (Visser et al., 2021).

Latin America has increasingly engaged with these global debates while simultaneously building its own mechanisms to foster research quality and visibility. Peru provides an illustrative case. Peruvian National Registry of Science, Technology and Innovation (RENACYT), administered by CONCYTEC, serves as the backbone of the national system for accrediting researchers. The 2022 regulation established a classification system based on a point scheme that assigns researchers to levels ranging from VII to Distinguished, considering academic degrees, publications indexed in Scopus or Web of Science, thesis supervision, and patents (CONCYTEC, 2022). This framework has helped systematize the recognition of researchers and align them with international databases, representing an important step in consolidating Peru's scientific ecosystem.

Nevertheless, significant limitations remain. The system privileges the accumulation of outputs over qualitative or leadership indicators, allowing publications in lower quartiles or conference proceedings to earn points at the same level as more influential contributions. RENACYT does not account for authorship position or corresponding author status, thereby omitting recognition of intellectual leadership within research teams. Although the regulation mentions obligations regarding integrity, these are not translated into measurable criteria such as monitoring retractions or ensuring compliance with ethical standards. Furthermore, the framework applies uniform scoring across disciplines, disregarding the diversity of publication practices in fields such as the humanities and biomedical sciences. As a result, the interaction between production, impact, leadership, and integrity, here approximated through retractions, remains only partially captured, limiting the system's ability to reflect the multidimensional nature of scientific performance. These omissions risk incentivizing quantity over meaningful contribution, replicating the same flaws that global initiatives like DORA, the Leiden Manifesto, and CoARA have sought to correct.

Peruvian universities increasingly rely on RENACYT accreditation to distribute incentives, promotions, and research support, giving the system particularly deep influence. However, if evaluation criteria remain predominantly quantitative, the national scientific culture risks being shaped toward opportunistic publication strategies rather than fostering integrity, originality, and leadership. This situation is critical given the current landscape of Peru's scientific production. Between 2000 and 2019, Peru's scientific output grew at an average annual rate of 13.6%, reaching a total of 24,482 publications (Mendoza-Chuctaya et al., 2021). Yet this growth conceals fragilities: only half of these publications had Peruvian affiliation, and 70% were concentrated in clinical medicine and biomedical sciences (Mendoza-Chuctaya et al., 2021), suggesting limited autonomy and strategic diversification of research. Evaluation processes have proven inadequate, as policies implemented by Peruvian universities have not significantly influenced production indexed in international databases (Millones-Gómez et al., 2021). The RENACYT scoring system exacerbates this problem by establishing strictly metric-based requirements that directly shape incentives and academic priorities (Barrutia Barreto et al., 2020). Despite national funding from CONCYTEC, experimental research capacity remains limited, as shown by the publication of only nine experimental COVID-19 studies during the pandemic, reflecting insufficient funding and a small pool of researchers (Coronel-Monje et al., 2023). International collaboration, particularly with the United States and Brazil, produces publications with higher citation rates (Mendoza-Chuctaya et al., 2021), but such dependence may perpetuate Peru's peripheral role in global knowledge production.

Taken together, these patterns depict a scientific ecosystem where quantitative expansion has not translated into qualitative maturation, illustrating how current evaluation frameworks may reinforce structural weaknesses rather than foster sustainable development. Against this backdrop, the present study adopts an integrative perspective by jointly analyzing production, journal-based impact (quartiles), leadership (corresponding authorship), and integrity (retractions) as interrelated—though imperfectly captured—dimensions of research performance. By situating RENACYT within the global movement for responsible metrics, this work seeks to contribute to the debate on how emerging scientific systems can reform their evaluation frameworks to support excellence and integrity in research.

## 2 Materials and methods

This study was designed as an observational, non-experimental, and analytical study, with the central purpose of generating empirical evidence to support proposals for responsible evaluation of RENACYT. Bibliometric indicators were not employed as an end in themselves, but rather as tools to identify limitations, gaps, and inconsistencies in the current framework for classifying researchers, in line with international principles of responsible research assessment.

The study population comprised all 11,789 researchers registered in RENACYT as of June 9, 2025. Following a data-cleaning process, researchers were excluded if there was no reliable correspondence between their RENACYT identifier and Scopus author profiles, or if the national registry listed identifiers that did not match active Scopus profiles. This exclusion process accounted for 2,138 cases (18.1% of the initial dataset), primarily due to missing, inconsistent, or non-traceable identifiers that could compromise bibliometric linkage. This process reduced the analytic sample to 9,651 researchers, ensuring traceability and validity of the bibliometric indicators employed.

Data were obtained from three complementary sources. RENACYT provided information on researcher classification levels (from VII to Distinguished), while Scopus and SciVal were used to extract individual-level publication and bibliographic metrics. The linkage process included the consolidation of diferent author profiles, and manual verification of homonyms to minimize biases caused by fragmented publication trajectories. The observation period covered 2019–2024, allowing for both cross-sectional comparisons and longitudinal trend analysis over 6 years.

Four main dimensions guided the analysis: scientific production, impact, leadership, and integrity. Production was measured as the total number of publications per year, enabling the evaluation of productivity trajectories and their distribution across RENACYT levels.

Impact was approximated using journal quartiles (Q1–Q4) according to the SCImago Journal Rank (SJR), providing an indicator of journal impact rather than direct article-level impact. This approach captures impact through the relative position of the journals in which researchers publish, with SJR quartiles reflecting prestige-weighted citation influence within each field (Falagas et al., 2008; González-Pereira et al., 2010).

Leadership was operationalized through corresponding authorship, given its role as the primary point of accountability in the publication process, responsible for coordinating the manuscript, managing interactions with editors and reviewers, and representing the study externally. This position reflects an operational form of leadership grounded in responsibility and coordination within research teams, making it a consistent and observable proxy in large-scale bibliometric analyses (He et al., 2021; Liu and Fang, 2014). Moreover, its distinction from other authorship roles—such as first or senior author—lies in its formal association with communication, validation, and stewardship of the research output, reinforcing its relevance as an indicator of scientific leadership (De Vanz et al., 2025).

Integrity was assessed by identifying retracted publications during the study period, thus incorporating a concrete indicator of risks to the quality and credibility of scientific output. Retractions were considered as observable signals within the scientific self-correction process, as they reflect the removal of publications affected by errors, ethical issues, or methodological concerns, thereby contributing to the reliability of the scholarly record (Koo and Lin, 2024; Okyay et al., 2025). Rather than being interpreted as direct evidence of misconduct, retractions were used to capture differential exposure to integrity-related risks across researchers, particularly in contexts of high publication pressure.

The analysis was conducted in three stages. First, descriptive statistics were applied to characterize the distribution of variables by RENACYT level, including means, standard deviations, and extreme values. This stage allowed for the identification of internal heterogeneity, outliers, and excess zeros, all relevant to evaluating the robustness of the classification system. Second, comparative analyses were carried out using analysis of variance (ANOVA) and multivariate analysis of variance (MANOVA), supplemented with Tukey *post-hoc* tests, in order to identify significant differences in productivity and impact between levels. Repeated-measures analyses were performed to examine annual production trends and to detect differentiated trajectories across levels, with Mauchly's test of sphericity used to verify assumptions and corrections applied as needed.

Third, regression analyses were conducted using Poisson and negative binomial (NB) models to estimate total scientific production and integrity (retractions). In addition, zero-inflated Poisson (ZIP) and zero-inflated negative binomial (ZINB) models were applied to the dimensions of impact (quartile-based outputs) and leadership (corresponding authorship), with the aim of uncovering patterns not explicitly recognized by the current RENACYT classification. Robust standard errors were employed to ensure valid statistical inference. The use of count-based and zero-inflated models was motivated by the discrete nature of publication data and the presence of excess zeros across multiple dimensions.

Goodness-of-fit tests were conducted for Poisson models, and overdispersion was formally assessed using likelihood ratio (LR) tests comparing Poisson and negative binomial specifications. Model selection was based on Akaike Information Criterion (AIC) and Bayesian Information Criterion (BIC), with only the best-fitting models reported.

Analyses were performed using SPSS (version 28.0) and Stata (version 17), which enabled robust statistical modeling and the handling of large-scale bibliometric data. The methodological strategy was designed so that each statistical procedure would generate specific insights for reconsidering the evaluation framework. For instance, ANOVA and Tukey tests revealed that significant differences between levels did not always follow a linear progression; ZIP and ZINB models revealed the coexistence of high productivity alongside a substantial proportion of inactive researchers, even within upper levels (44.13, 47.40, 45.29, and 55.54% of researchers without publications in Q1, Q2, Q3, and Q4 journals, respectively); and the inclusion of leadership and integrity indicators highlighted dimensions absent from current regulations. Collectively, these approaches allowed the analysis to move beyond the purely accumulative logic of publication counts, illustrating how RENACYT may currently reward volume over quality and overlook meaningful contributions to science.

Ethical considerations were observed throughout. The study relied exclusively on secondary data from institutional and bibliographic sources, without any contact with human participants or collection of sensitive data. All results were reported in aggregate, ensuring that individual researchers could not be identified. The explicit inclusion of retractions as a variable reflected a deliberate commitment to align with international recommendations on transparency and accountability in research evaluation.

In sum, the methodological design did not merely describe the activity of RENACYT-registered researchers but sought to generate robust, comparative evidence that could serve as the basis for proposing reforms to strengthen the system's capacity to recognize quality, leadership, and integrity in research, within an integrated and multidimensional evaluation framework, moving beyond a narrow focus on publication counts.

## 3 Results

### 3.1 Overall production and annual trends

The analytic set comprises 9,651 researchers and 92,284 publications across 2019–2024. The cumulative mean output was 9.6 ± 16.0 publications per researcher, with highly skewed distributions. In all levels and years, minimum values remained at zero, while individual maxima were substantial (e.g., 334 in Distinguished, 247 in Level II, and 216 in Level V), confirming pronounced within-level dispersion (period SDs: Distinguished 51.0; I 25.4; II 19.7; VII 4.2).

Group sizes remained uneven (VII = 3,689; VI = 2,049; V = 1,419; IV = 963; III = 644; II = 484; I = 159; Distinguished = 245). Distinguished researchers represent 2.5% of personnel but contribute 16.6% of total output (15,306 publications), whereas Level VII accounts for 38.2% of researchers and 15.6% of output (14,425 publications). The cumulative gradient by level is preserved: Distinguished (62.5), I (40.4), II (24.6), III (15.9), IV (11.7), V (8.4), VI (5.2), VII (3.9). Tukey's test confirms that only Levels VI and VII do not differ significantly, while all other pairwise comparisons remain distinct (*F* = 1162.572; *p* < 0.001). These descriptive patterns are detailed in Table 1.

**Table 1:** Scientific production of RENACYT researchers, 2019–2024.

RENACYT level		2019	2020	2021	2022	2023	2024	Period^&^	
Distinguished researcher (*n* = 245)	Total	1,950	2,290	2,724	3,043	2,799	2,500	15,306	
	Mean	8.0	9.3	11.1	12.4	11.4	10.2	62.5	G
	SD	7.8	8.6	9.9	11.6	12.2	10.2	51.0	
	Min	0	0	0	0	0	0	1	
	Max	54	56	61	61	76	63	334	
I (*n* = 159)	Total	533	764	958	1,331	1,547	1,252	6,385	
	Mean	3.4	4.8	6.1	8.4	9.8	7.9	40.4	F
	SD	3.4	4.0	4.8	7.0	10.2	7.7	25.4	
	Min	0	0	0	0	0	0	3	
	Max	23	20	24	35	66	53	129	
II (*n* = 484)	Total	1,181	1,670	1,971	2,243	2,649	2,183	11,897	
	Mean	2.4	3.5	4.1	4.6	5.5	4.5	24.6	E
	SD	3.9	5.3	3.8	4.4	5.9	4.3	19.7	
	Min	0	0	0	0	0	0	0	
	Max	67	96	28	34	52	29	247	
III (*n* = 644)	Total	905	1,217	1,596	1,874	2,395	2,276	10,263	
	Mean	1.4	1.9	2.5	2.9	3.7	3.5	15.9	D
	SD	2.0	2.4	2.6	2.6	4.0	4.0	10.5	
	Min	0	0	0	0	0	0	0	
	Max	22	19	19	17	35	32	83	
IV (*n* = 963)	Total	967	1,352	1,852	2,101	2,508	2,504	11,284	
	Mean	1.0	1.4	1.9	2.2	2.6	2.6	11.7	C
	SD	1.7	1.9	2.2	2.4	3.0	3.1	8.6	
	Min	0	0	0	0	0	0	0	
	Max	16	24	15	28	29	33	93	
V (*n* = 1,419)	Total	905	1,309	1,782	2,335	2,914	2,727	11,972.00	
	Mean	0.6	0.9	1.3	1.6	2.1	1.9	8.4	B
	SD	1.5	1.6	1.9	2.6	2.9	2.6	9.7	
	Min	0	0	0	0	0	0	0	
	Max	38	23	34	39	44	41	216	
VI (*n* = 2,049)	Total	671	1,140	1,690	2,163	2,625	2,463	10,752	
	Mean	0.3	0.6	0.8	1.1	1.3	1.2	5.2	A
	SD	0.9	1.2	1.6	2.1	2.0	1.9	6.2	
	Min	0	0	0	0	0	0	0	
	Max	13	20	25	47	24	31	125	
VII (*n* = 3,689)	Total	719	1,388	1,893	2,719	3,772	3,934	14,425	
	Mean	0.2	0.4	0.5	0.7	1.0	1.1	3.9	A
	SD	0.7	1.0	1.1	1.4	1.6	1.6	4.2	
	Min	0	0	0	0	0	0	0	
	Max	14	19	22	30	26	29	79	
Total (*n* = 9,651)	Total	7,831	11,130	14,466	17,809	21,209	19,839	92,284	
	Mean	0.8	1.2	1.5	1.8	2.2	2.1	9.6	
	SD	2.3	2.8	3.1	3.7	4.1	3.5	16.0	
	Min	0	0	0	0	0	0	0	
	Max	67	96	61	61	76	63	334	
ANOVA	F							1,162.572	
	p							0.000	

^&^Tukey's univariate test.

At the aggregate level, annual production increased from 7,831 (2019) to 21,209 (2023) and declined slightly to 19,839 in 2024 (−6.5%). Mean output per researcher followed a similar trajectory, rising from 0.8 (2019) to 2.2 (2023) and decreasing to 2.1 (2024). Most levels peaked in 2023 (e.g., V = 2,914; VI = 2,625; IV = 2,508), while Distinguished reached its maximum in 2022 (3,043) and declined thereafter. In contrast, Level VII continued increasing and peaked in 2024 (3,934). Yearly maxima varied across levels, reinforcing the high internal heterogeneity observed.

To examine temporal dynamics, we tested whether annual output changed over time and whether trajectories differed across RENACYT levels using a repeated-measures framework. The multivariate tests and within-subject effects summarized in Table 2 indicate that both Time and the Time × Level interaction are highly significant across all specifications (all *p* < 0.001). Specifically, Table 2 shows significant multivariate effects for Time (*F* = 497.566; *p* < 0.001) and Time × Level (Pillai's trace, Wilks' lambda, and Hotelling's trace: *F* = 35.000; *p* < 0.001), confirming that production changed over time and that the magnitude of this change differed by RENACYT level.

**Table 2:** Repeated-measures analysis of annual production, 2019–2024.

	Factor	Test	*F*	*p*
Multivariate tests	Time	Pillai's trace	497.566	0.000
		Wilks' lambda	497.566	0.000
		Hotelling's trace	497.566	0.000
		Roy's largest root	497.566	0.000
	Time x Level	Pillai's trace	35.000	0.000
		Wilks' lambda	35.000	0.000
		Hotelling's trace	35.000	0.000
		Roy's largest root	7.000	0.000
Tests of within-subjects effects	Time	Sphericity Assumed	771.392	0.000
		Greenhouse-Geisser	771.392	0.000
		Huynh-Feldt	771.392	0.000
		Lower-bound	771.392	0.000
	Time x Level	Sphericity Assumed	44.662	0.000
		Greenhouse-Geisser	44.662	0.000
		Huynh-Feldt	44.662	0.000
		Lower-bound	44.662	0.000
Tests of within-subjects contrasts	Time	Linear	1,583.547	0.000
		Quadratic	534.300	0.000
		Cubic	210.294	0.000
		4th order	22.295	0.000
		5th order	0.008	0.930
	Time x Level	Linear	64.414	0.000
		Quadratic	71.100	0.000
		Cubic	18.681	0.000
		4th order	11.062	0.000
		5th order	3.8749	0.000

Mauchly's test was significant (*W* = 0.349; χ^2^ = 10,136.874; *p* < 0.001), indicating violation of sphericity and supporting the use of robust corrections (Greenhouse–Geisser and Huynh–Feldt). Consistent with Table 2, within-subject effects confirm a strong temporal effect (*F* = 771.392; *p* < 0.001) and a significant interaction between time and level (*F* = 44.662; *p* < 0.001). Polynomial contrasts show that temporal dynamics are best described up to a fourth-order component (linear, quadratic, cubic, and fourth order all *p* < 0.001), while the fifth-order term is not significant (*p* = 0.930). In contrast, the Time × Level interaction remains significant across all polynomial orders, including the fifth, indicating more complex and differentiated trajectories when disaggregated by level.

**Figure 1:**
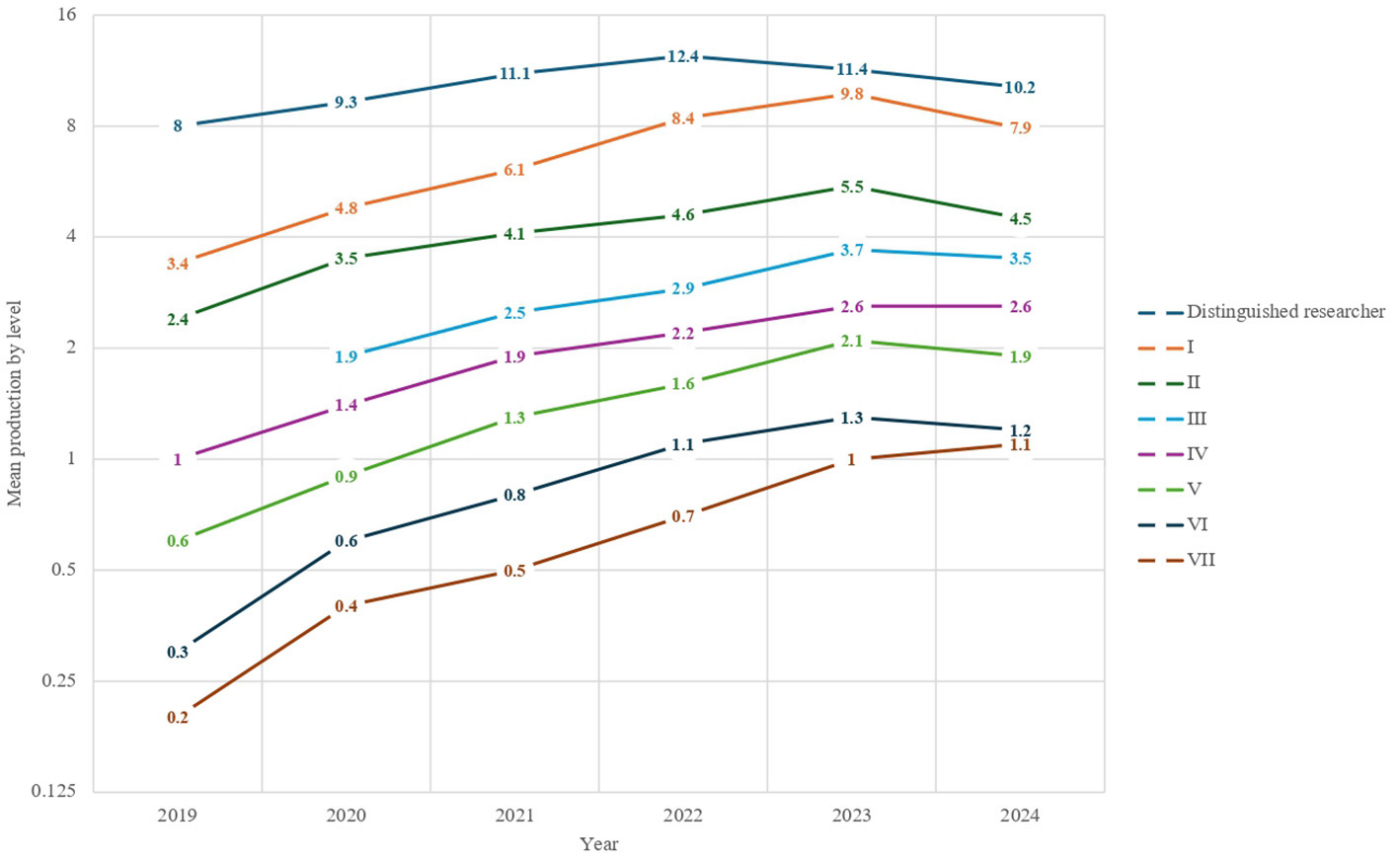
Mean annual scientific production by RENACYT level, 2019-2024. Distinguished researchers peaked in 2022 (mean = 12.4), most intermediate levels in 2023, and Level VII showed a sustained increase through 2024.

Figure 1 illustrates the evolution of mean annual production by level over 2019–2024, highlighting clear heterogeneity in temporal patterns. Aggregate production increased from 7,831 in 2019 to 21,209 in 2023, followed by a slight decline to 19,839 in 2024 (−6.5%). Mean output per researcher followed a similar pattern, rising from 0.8 to 2.2 and then decreasing marginally to 2.1.

However, these trends are not uniform across RENACYT levels. Most levels—particularly IV, V, and VI—reached their production peak in 2023 (e.g., V = 2,914; VI = 2,625; IV = 2,508). In contrast, the Distinguished level peaked earlier, in 2022 (3,043), and declined thereafter, suggesting that even the most productive group is not immune to temporal contraction. Conversely, Level VII exhibits a sustained upward trajectory, reaching its maximum in 2024 (3,934). These divergent patterns reinforce the presence of structurally differentiated trajectories across levels.

Finally, to assess whether cumulative differences across RENACYT levels remain statistically robust under a count-data framework, total scientific production was modeled using both Poisson and negative binomial regression, with Level VII as the reference category.

As shown in Table 3, coefficients are positive and highly significant across the entire gradient in both models. In the Poisson model, the coefficients were 0.305 for Level VI, 0.769 for Level V, 1.097 for Level IV, 1.405 for Level III, 1.838 for Level II, 2.336 for Level I, and 2.771 for Distinguished researchers. In the negative binomial model, the corresponding coefficients were 0.294 for Level VI, 0.769 for Level V, 1.097 for Level IV, 1.405 for Level III, 1.838 for Level II, 2.336 for Level I, and 2.771 for Distinguished researchers; all coefficients were statistically significant (*p* < 0.001). These results indicate a clear monotonic increase in expected production as the level rises, fully consistent with the cumulative patterns observed in Table 1.

**Table 3:** Poisson and negative binomial regression models of total scientific production, 2019–2024.

RENACYT level	Poisson regression			Negative binomial regression		
	Coef.	*z*	*p*	Coef.	*z*	*p*
Distinguished	2.771	238.80	0.000	2.771	63.80	0.000
I	2.336	155.38	0.000	2.336	43.50	0.000
II	1.838	148.44	0.000	1.838	55.58	0.000
III	1.405	108.80	0.000	1.405	46.81	0.000
IV	1.097	87.33	0.000	1.097	41.93	0.000
V	0.769	62.20	0.000	0.769	32.92	0.000
VI	0.305	24.03	0.000	0.294	13.60	0.000
Cons	1.364	163.77	0.000	1.364	102.08	0.000

Although Poisson and negative binomial estimates were highly consistent, goodness-of-fit tests indicated that the Poisson model did not adequately fit the data. Deviance and Pearson goodness-of-fit tests were significant (both *p* < 0.001), and the likelihood-ratio test confirmed the presence of overdispersion (*p* < 0.001). Therefore, the negative binomial specification was retained as the more appropriate model. Its AIC and BIC values were 56,076.9 and 56,141.5, respectively.

Notably, the model identifies a significant difference between Levels VI and VII in both specifications, although with different estimates and test statistics: Poisson coef. = 0.305, *z* = 24.03, *p* < 0.001; negative binomial coef. = 0.294, *z* = 13.60, *p* < 0.001. This occurs despite thier lack of statiscal separation in Tukey's test. This suggests that the regression approach is more sensitive to differences in count distributions, particularly in lower productivity strata. Overall, the findings confirm that RENACYT levels effectively capture broad productivity gradients, while also evidencing residual heterogeneity within levels that is not fully resolved by categorical classification.

### 3.2 Differentiation by journal quality (Q1–Q4)

The distribution of publications across journals classified by quartile (Q1–Q4) reveals differentiated patterns across RENACYT levels. As shown in Table 4, higher levels continue to concentrate a disproportionate share of production in high-impact journals (Q1 and Q2), whereas lower levels maintain a smaller but non-zero presence. This pattern confirms that the RENACYT hierarchy captures gradients of visibility and journal quality, although important limitations remain.

**Table 4:** Scientific production of RENACYT researchers by journal quartile, 2019–2024.

RENACYT level		Q1^&^	Statistic	Q2^&^		Q3^&^		Q4^&^	
Distinguished level (*n* = 245)	Total	7,547		2,882		1,842		1,523	
	Mean	30.8	F	11.8	G	7.5	F	6.2	G
	SD	31.6		12.4		11.4		13.3	
	Min	1		0		0		0	
	Max	252		94		86		129	
I (*n* = 159)	Total	2,333		1,165		1,131		909	
	Mean	14.8	E	7.4	F	7.2	F	5.8	G
	SD	10.1		6.6		10.3		9.7	
	Min	0		0		0		0	
	Max	62		38		59		44	
II (*n* = 484)	Total	4,115		2,136		2,035		1,673	
	Mean	8.5	D	4.4	E	4.2	E	3.5	F
	SD	6.9		4.4		5.1		6.9	
	Min	0		0		0		0	
	Max	47		27		31		58	
III (*n* = 644)	Total	3,119		1,902		1,973		1,505	
	Mean	4.8	C	3.0	D	3.1	D	2.3	DE
	SD	4.2		3.0		3.4		4.0	
	Min	0		0		0		0	
	Max	27		29		18		31	
IV (*n* = 963)	Total	3,033		1,988		2,137		1,939	
	Mean	3.1	B	2.1	C	2.2	C	2.0	CD
	SD	3.5		2.3		2.6		3.5	
	Min	0		0		0		0	
	Max	51		31		16		36	
V (*n* = 1,419)	Total	2,548		1,802		2,114		2,050	
	Mean	1.8	A	1.3	B	1.5	B	1.4	BC
	SD	2.2		1.6		1.9		2.5	
	Min	0		0		0		0	
	Max	24		14		19		38	
VI (*n* = 2,049)	Total	1,999		1,417		2,170		1,879	
	Mean	1.0	A	0.7	A	1.1	AB	0.9	AB
	SD	1.6		1.1		2.1		1.9	
	Min	0		0		0		0	
	Max	15		20		48		32	
VII (*n* = 3,689)	Total	2,626		1,975		2,583		2,605	
	Mean	0.7	A	0.5	A	0.7	A	0.7	A
	SD	1.3		0.9		1.2		1.5	
	Min	0		0		0		0	
	Max	34		14		17		27	
Total (*n* = 9,651)	Total	27,320		15,267		15,985		14,083	
	Mean	2.8		1.6		1.7		1.5	
	SD	7.8		3.5		3.5		3.8	
	Min	0		0		0		0	
	Max	252		94		86		129	
ANOVA	F	1,090.183		780.758		324.633		150.524	
	p	0.000		0.000		0.000		0.000	
MANOVA^#^	F	227.416		308.698		410.342		1,600.630	
	p	0.000		0.000		0.000		0.000	

^&^Tukey's univariate test. ^#^Multivariate tests: Pillai's trace, Wilks' lambda, Hotelling's trace, and Roy's largest root.

In Q1, Distinguished researchers averaged 30.8 articles per author, well above Level I (14.8) and the remaining levels, declining to 0.7 in Level VII. Dispersion remained substantial in upper strata (SD = 31.6 in Distinguished), with maxima reaching 252 publications, while lower levels rarely exceeded one Q1 article per researcher on average. In Q2, the pattern was similar: Distinguished researchers averaged 11.8 articles and Level I 7.4, whereas Levels VI and VII remained below one article per researcher.

By contrast, differences became less pronounced in Q3 and Q4. Distinguished and Level I remained in the same Tukey group in both quartiles, with similar averages in Q3 (7.5 vs. 7.2) and Q4 (6.2 vs. 5.8), indicating convergence in mid- and lower-tier journals. Thus, although the upper strata dominate high-impact output, the distinction between levels weakens outside Q1–Q2.

Analysis of variance supports this pattern, with highly significant differences across all quartiles (Q1: *F* = 1,090.183; Q2: *F* = 780.758; Q3: *F* = 324.633; Q4: *F* = 150.524; all *p* < 0.001). Multivariate results were likewise significant across specifications (all *p* < 0.001), confirming that publication profiles differ systematically between levels. Taken together, these findings indicate that RENACYT differentiates more clearly in the upper quartiles than in the intermediate and lower ones, which is especially relevant for evaluating how the system interprets journal quality as a marker of performance.

Nevertheless, descriptive analysis alone is insufficient to capture the magnitude of these differences, as a substantial proportion of researchers produced no publications in certain quartiles, generating excess zeros. To address this, zero-inflated Poisson (ZIP) and zero-inflated negative binomial (ZINB) regression models were applied.

As shown in Table 5, and using Level VII as the reference category, all higher RENACYT levels exhibit positive and statistically significant coefficients across quartiles (all *p* < 0.001), confirming a consistent gradient of increasing publication. In Q1, Distinguished researchers show a coefficient of 2.716, followed by Level I (2.413) and Level II (2.102), indicating strong differences relative to the baseline.

**Table 5:** Zero-inflated and negative binomial regression of RENACYT researchers' output by quartile.

RENACYT Level	Q1		Q2		Q3		Q4	
	Coef.	*p*	Coef.	*p*	Coef.	*p*	Coef.	*p*
Production	0.014	0.000	0.020	0.000	0.031	0.000	0.054	0.000
RENACYT level								
Distinguished	2.716	0.000	1.582	0.000	0.194	0.094	−1.647	0.000
I	2.413	0.000	1.644	0.000	0.736	0.000	−0.703	0.000
II	2.102	0.000	1.502	0.000	0.850	0.000	−0.236	0.010
III	1.657	0.000	1.303	0.000	0.909	0.000	0.096	0.187
IV	1.309	0.000	1.073	0.000	0.772	0.000	0.246	0.000
V	0.840	0.000	0.692	0.000	0.501	0.000	0.235	0.000
VI	0.415	0.000	0.209	0.000	0.301	0.000	0.061	0.238
Cons	−0.299	0.000	−0.543	0.000	−0.350	0.000	−0.357	0.000
Inflate								
Production	−1.253	0.000	−1.207	0.000	−1.437	0.000	−1.102	0.000
RENACYT level								
Distinguished	−23.299	0.000	1.653	0.044	1.632	0.067	4.984	0.006
I	3.552	0.137	4.771	0.054	7.963	0.016	9.572	0.000
II	2.736	0.000	3.469	0.001	−1.468	0.170	0.377	0.788
III	0.739	0.169	1.978	0.004	1.408	0.143	1.792	0.011
IV	1.520	0.000	2.132	0.000	1.755	0.001	−0.238	0.625
V	1.610	0.000	1.324	0.000	0.360	0.352	0.568	0.071
VI	1.597	0.000	0.287	0.177	0.133	0.530	−0.093	0.660
Cons	0.555	0.022	1.176	0.000	1.479	0.000	1.969	0.000

Model comparison favored the ZINB specification, which accounts simultaneously for overdispersion and excess zeros. The AIC and BIC values for the ZINB models were, respectively, 32,829.7 and 32,966.0 for Q1, 26,913.6 and 27,049.9 for Q2, 29,333.6 and 29,469.9 for Q3, and 26,493.8 and 26,630.1 for Q4. ZIP models yielded higher AIC and BIC values for Q1–Q3, while no convergent ZIP solution was obtained for Q4.

The inflation component provides additional insight into the probability of zero production. Across quartiles, the direction and significance of coefficients vary by level, indicating that the probability of zero publications does not decrease monotonically with the RENACYT hierarchy.

In Q2 and Q3, the pattern becomes more heterogeneous. While the count component remains strongly positive and significant, the inflation model shows positive and significant coefficients for higher levels (e.g., Distinguished: 1.430 in Q2 and 2.428 in Q3), indicating that the probability of zero publications does not decrease monotonically with level. Instead, zero inflation persists even among upper strata, suggesting coexistence of productive and inactive subgroups within the same category. Additionally, some coefficients lose significance in intermediate levels (e.g., IV and V in Q3), reinforcing the presence of internal variability.

In Q4, instead of a separate Poisson specification, the ZINB model was retained. Results show that several upper levels (including Distinguished, Level I, and Level II) exhibit negative and statistically significant coefficients in the count component, indicating that, after controlling for total production, higher-ranked researchers are comparatively less oriented toward lower-quartile journals. This finding complements the descriptive results in Table 4, where these groups still accumulate Q4 publications in absolute terms but exhibit a stronger concentration in Q1 and Q2 journals.

Overall, the ZINB models reveal a dual structure: while the RENACYT hierarchy captures a clear gradient in publication intensity, it does not eliminate within-level heterogeneity in zero production. This is particularly evident outside Q1, where the probability of non-publication remains non-negligible even among higher levels. These findings suggest that the classification system differentiates productivity more effectively than participation, especially in mid- and lower-impact journals.

### 3.3 Recognition of scientific leadership through corresponding authorship

Leadership within research teams was examined through corresponding authorship, considered an indicator of intellectual responsibility in publications. Results from the zero-inflated Poisson regression (Table 6) show that, relative to Level VII, all higher RENACYT levels display positive and highly significant coefficients (all *p* < 0.001), indicating a greater likelihood of assuming corresponding authorship as one moves up the hierarchy.

**Table 6:** Zero-inflated poisson and negative binomial regression models of corresponding authorships among RENACYT researchers.

RENACYT Level	Zero-inflated poisson regression			Zero-inflated negative binomial regression		
	Coef.	*z*	*p*	Coef.	*z*	*p*
Production	0.012	6.31	0.000	0.034	16.43	0.000
RENACYT level						
Distinguished	1.535	9.41	0.000	0.918	7.85	0.000
I	1.624	13.20	0.000	1.156	10.51	0.000
II	1.384	14.91	0.000	1.226	16.41	0.000
III	1.033	13.77	0.000	1.050	15.69	0.000
IV	0.826	11.71	0.000	0.868	14.13	0.000
V	0.667	6.55	0.000	0.612	10.38	0.000
VI	0.301	3.74	0.000	0.329	5.22	0.000
Cons	0.363	6.30	0.000	−0.376	−7.76	0.000
Inflate						
Production	−0.094	−7.59	0.000	−0.706	−8.62	0.000
Level						
Distinguished	0.739	2.14	0.032	1.514	1.51	0.131
I	−0.131	−0.38	0.701	3.367	3.11	0.002
II	−0.551	−2.82	0.005	0.846	1.86	0.062
III	−0.520	−3.38	0.001	1.357	2.88	0.004
IV	−0.397	−3.32	0.001	1.055	3.57	0.000
V	−0.372	−3.85	0.000	0.144	0.62	0.533
VI	−0.077	−0.91	0.362	0.314	1.86	0.062
Cons	0.860	11.46	0.000	1.375	8.20	0.000

For instance, Level I researchers exhibit a coefficient of 1.624, slightly exceeding that of Distinguished researchers (1.535), suggesting that leadership roles are not exclusively concentrated in the highest category. Instead, leadership appears distributed across upper levels rather than strictly monopolized by the elite.

The inflation component adds a relevant nuance. While overall production reduces the probability of zero corresponding authorships (coef. = −0.094; *p* < 0.001), patterns across levels are heterogeneous. Distinguished researchers show a positive and significant coefficient (0.739; *p* = 0.004), indicating that even at the highest level there exists a subgroup that does not assume corresponding authorship. In contrast, several intermediate levels (II–V) display negative and significant coefficients, reflecting a lower likelihood of zero leadership roles relative to the reference category, while others (Level I and VI) are not statistically different.

Taken together, these findings indicate that the RENACYT system captures a general gradient of leadership, but not in a strictly hierarchical or uniform manner. While higher levels are more likely to assume corresponding authorship, the presence of non-significant and even positive inflation coefficients reveals internal heterogeneity, suggesting that leadership is not consistently exercised within levels. This points to limitations in using categorical ranking alone to infer scientific responsibility and leadership roles.

To account for overdispersion and excess zeros, both zero-inflated Poisson (ZIP) and zero-inflated negative binomial (ZINB) models were estimated. Model comparison favored the ZINB specification, which provided a better fit to the data. The AIC and BIC values for the ZINB model were 28,580.1 and 28,716.4, respectively, compared with higher values for the ZIP model, confirming the superiority of the ZINB approach.

### 3.4 Risks to scientific integrity from retractions

The analysis of retractions provides a critical perspective on scientific integrity, as it directly reflects problematic publication outcomes. In this specification, Distinguished researchers are used as the reference category due to instability and sparse counts when lower levels were previously used as the baseline.

As shown in Table 7, scientific production is positively and significantly associated with retractions (coef. = 0.013; *p* < 0.001), indicating that higher output increases the likelihood of accumulating at least one retracted publication.

**Table 7:** Poisson and negative binomial regression models of retractions among RENACYT researchers.

RENACYT Level	Poisson regression			Negative binomial regression		
	Coef.	*z*	*p*	Coef.	*z*	*p*
Production	0.014	4.61	0.000	0.035	3.67	0.000
RENACYT level						
I	0.433	0.43	0.669	0.279	0.39	0.698
II	−1.450	−1.92	0.055	−0.457	−0.53	0.598
III	−1.566	−1.71	0.088	−0.354	−0.33	0.739
IV	−1.908	−2.02	0.044	−0.657	−0.58	0.564
V	−1.749	−2.47	0.013	−0.412	−0.49	0.626
VI	−3.670	−3.21	0.001	−2.214	−1.76	0.079
VII	−4.238	−3.71	0.000	−2.730	−2.17	0.030
Cons	−4.030	−7.13	0.000	−5.635	−6.79	0.000

Relative to Distinguished researchers, most RENACYT levels display negative and statistically significant coefficients (Levels II–VII; *p* < 0.05), indicating a lower probability of retractions compared to the highest level. In contrast, Level I does not differ significantly (*p* = 0.372), suggesting a similar risk profile to the elite group.

These results indicate that retractions are not uniformly distributed across the hierarchy but are disproportionately concentrated at the top. Rather than increasing monotonically with level, the pattern reveals a concentration effect, where the highest strata accumulate greater exposure to retraction events.

However, model diagnostics indicate that the Poisson specification is affected by overdispersion (LR test, *p* < 0.001), which may bias coefficient estimates and standard errors. When overdispersion is accounted for using the negative binomial model, the pattern becomes less pronounced. In this specification, most differences between RENACYT levels and Distinguished researchers lose statistical significance, and only Level VII remains significantly lower (β = −2.730; *p* = 0.030), while Level VI approaches significance (*p* = 0.079).

The AIC and BIC values for the negative binomial model (355.2 and 427.0, respectively) confirm a better fit compared to the Poisson specification, supporting its use as the preferred model.

These results suggest that retractions should not be interpreted as being structurally concentrated in the highest RENACYT levels, but rather as rare events associated with publication volume and exposure.

Overall, the findings highlight a structural tension: while higher levels are associated with greater productivity, they also exhibit higher exposure to integrity risks. However, this relationship is better explained by exposure effects than by systematic differences in misconduct across levels. This suggests that evaluation systems primarily based on output volume may inadvertently amplify the probability of problematic publications, emphasizing the need to integrate quality and integrity-sensitive indicators into research assessment frameworks.

## 4 Discussion

This study provides a critical evaluation of RENACYT, implemented by CONCYTEC. The findings confirm that higher levels concentrate more output and have greater presence in Q1–Q2 journals, indicating that the system partially captures a performance gradient. However, the coexistence of highly productive researchers alongside inactive ones—even within the top levels—demonstrates that RENACYT fails to adequately differentiate between sustained trajectories and episodic productivity. This pattern is further supported by the zero-inflated models, which reveal that non-publication persists even among higher levels outside Q1. This evidence suggests that the system captures intensity of production more effectively than continuity of participation, a limitation that echoes what has been described elsewhere as an “iron cage effect,” in which rigid metrics standardize academic evaluation at the expense of diversity and autonomy (Jin and Jiang, 2024; Lambovska, 2023; Rowlands and Wright, 2021).

The distribution of publications by quartiles reinforces this ambivalence. The fact that Distinguished researchers average high Q1 outputs confirms the existence of a Peruvian elite with international visibility. Yet, the statistical convergence across levels in Q3–Q4 indicates that the system does not effectively discriminate in the intermediate strata. This convergence is not only descriptive but also reflected in regression results, where differences across levels become less structurally consistent in mid- and lower-tier quartiles. Moreover, count models that account for overdispersion and excess zeros (ZINB) show that, after controlling for total production, higher-ranked researchers are comparatively less oriented toward lower-quartile journals, reinforcing the idea of differentiated publication strategies rather than uniform performance gradients. These findings highlight that journal quartiles capture relative positioning in publication strategies rather than intrinsic research quality, which is consistent with international critiques against the mechanical use of journal metrics, as emphasized by the Declaration on Research Assessment (DORA; Cagan, 2013) and the Leiden Manifesto (Hicks et al., 2015). Studies have shown that the institutionalization of “impact” has reshaped academic discourse (Kochetkov, 2024; Wróblewska, 2021), but it has also encouraged undesirable practices such as article fragmentation, inflated coauthorship, and opportunistic citation strategies (Fire and Guestrin, 2018; Sandström and van den Besselaar, 2016).

Leadership, approximated through corresponding authorship, provides an additional nuance. Although higher levels show a greater probability of assuming this role, subgroups within the elite fail to exercise it, questioning the assumed link between hierarchy and intellectual direction. Notably, the absence of corresponding authorship remains statistically present even in the highest level, confirming that leadership is not uniformly distributed within categories. This interpretation is strengthened by zero-inflated models that account for heterogeneous participation, showing that even within upper levels, the probability of non-participation in leadership roles remains non-negligible. This reinforces the interpretation of corresponding authorship as an operational, rather than exhaustive, indicator of leadership, capturing formal responsibility but not the full spectrum of intellectual contribution. This finding resonates with recommendations from the Coalition for Advancing Research Assessment (CoARA), which calls for recognition of diverse contributions—including mentorship, team coordination, and data management—that remain invisible in traditional metrics (CoARA, 2024; Smit and Hessels, 2021; Thomas et al., 2020).

The dimension of integrity offers perhaps the most critical signal. Although the absolute number of retractions is low, initial Poisson-based results suggest a higher exposure among upper levels, particularly Distinguished and Level I, however, once overdispersion is accounted for through negative binomial models, these differences become substantially attenuated, with only the lowest level (VII) remaining significantly distinct. This shift across model specifications underscores the importance of appropriate count-data modeling when interpreting rare events such as retractions. These are not “high rates,” but rather a concentration of risk that shows how pressure to comply with quantitative criteria exposes researchers to ethical and quality concerns. Importantly, this pattern is consistent with an exposure-driven mechanism, where higher productivity increases the probability of accumulating at least one retraction event. Therefore, retractions should not be interpreted as indicators of misconduct concentrated in specific levels, but as rare events linked to publication volume and structural exposure. This interpretation is consistent with recent international approaches such as the SCImago IRIS framework, which treats retractions not as direct evidence of misconduct but as early signals of differential exposure to integrity risks, requiring contextual and qualitative interpretation rather than punitive conclusions. This pattern aligns with Goodhart's Law, which holds that once an indicator becomes a target, it ceases to be a valid measure (Fire and Guestrin, 2018). Editorial literature has also warned that unchecked growth in publications can saturate peer-review systems and compromise rigor (Montazerian and Dorch, 2022; Quaderi, 2023). Experiences such as Italy's selective funding linked to national evaluations illustrate how strict reliance on metrics can produce inequalities and unintended effects (Grisorio and Prota, 2020).

The Peruvian context makes these tensions visible. Between 2000 and 2019, national scientific production grew at an annual rate of 13.6%, yet 70% was concentrated in clinical medicine and biomedical sciences, signaling limited diversification (Mendoza-Chuctaya et al., 2021). During the pandemic, only nine experimental studies on COVID-19 were published, exposing structural weaknesses in research capacity (Coronel-Monje et al., 2023). At the university level, institutional policies have had limited impact on indexed production (Millones-Gómez et al., 2021), while the current RENACYT framework has incentivized quantitative accumulation strategies rather than innovation or integrity (Barrutia Barreto et al., 2020).

Methodologically, this study offers important strengths: the triangulation of RENACYT, Scopus, and SciVal data; rigorous cleaning and disambiguation of researcher profiles; and the use of count models that correct for overdispersion and excess zeros. The convergence of results across Poisson, negative binomial, and zero-inflated specifications further reinforces the robustness of the findings. These elements provide robustness and replicability to the findings. Nonetheless, limitations must be acknowledged. First, reliance on Scopus alone may underrepresent areas such as humanities and social sciences, where other databases capture output more effectively (Visser et al., 2021). Rather than a purely methodological constraint, this reflects structural differences in disciplinary publication cultures, which shape how productivity, impact, and visibility are expressed across fields. In this sense, the present study provides a system-level baseline that enables future research to disaggregate these patterns and develop discipline-sensitive evaluation frameworks. Second, corresponding authorship is an incomplete proxy for leadership, as it does not account for other forms of academic direction (e.g., mentorship or conceptualization). Third, the 6-year window (2019–2024) offers only a medium-term perspective, without capturing the effects of the most recent regulatory reforms.

The evidence underscores the need for a deep reform of the system. The RENACYT privileges the accumulation of quantitative products through a homogeneous scale across disciplines. As an alternative, we propose a hybrid model based on four pillars: (1) discipline-sensitive metrics, to avoid homogenization that obscures diverse practices (Smit and Hessels, 2021; Thomas et al., 2020); (2) explicit leadership indicators, including corresponding authorship, project direction, and roles defined by the CRediT taxonomy; (3) integrity metrics, such as monitoring retractions, sanctions for misconduct, and data transparency; and (4) qualitative narrative components, such as narrative CVs, societal impact, innovation, and mentorship (Cagan, 2013; CoARA, 2024). Importantly, integrity indicators should be interpreted within a risk-based and contextual framework, rather than as isolated signals of misconduct. In line with recent developments such as the IRIS framework, these integrity dimensions should be interpreted as risk-based signals—supported by normalized indicators and benchmarking—rather than as isolated metrics, reinforcing the need to combine quantitative evidence with institutional context and expert judgment.

This approach aligns with international experiences such as Mexico's Sistema Nacional de Investigadores (SNI), which combines metrics with peer review (CONAHCYT, 2023), and Colombia's Minciencias model, which integrates individual and group evaluations to foster collaborative networks (Minciencias, 2024).

Accordingly, the findings of this study should not be seen solely as a critique of RENACYT but as a contribution to the broader international debate on research assessment. The Peruvian case illustrates how developing countries, by adopting metric-driven models, reproduce tensions already observed in consolidated systems, but also hold the opportunity to innovate early with responsible evaluation frameworks. RENACYT thus becomes a case study that speaks to global experiences—such as the UK's Research Excellence Framework, Australia's ERA, and Mexico's SNI—and provides evidence on the risks and opportunities of national assessment. More than a local diagnosis, this article shows that building hybrid systems attentive to integrity, leadership, and societal impact is part of a shared agenda in contemporary science. In this sense, Peru offers the academic world a valuable lesson: even in resource-constrained settings, it is possible to design reforms that strengthen national research while also enriching the international debate on fairer, more transparent, and sustainable evaluation practices.

## Data Availability

The original contributions presented in the study are included in the article/Supplementary material, further inquiries can be directed to the corresponding author.
